# The safety and efficacy of the Mo.Ma system device for carotid artery stenting: A single-center experience from Taiwan

**DOI:** 10.3389/fcvm.2022.926513

**Published:** 2022-09-15

**Authors:** Cheng-Chung Cheng, Chin-Sheng Lin, Wei-Hsian Yin, Chin Lin, I-Fan Liu, Yu-Feng Lee, Wei-Ting Liu, Hao-Neng Fu, Chien-Lung Huang, Tien-Ping Tsao

**Affiliations:** ^1^Division of Cardiology, Department of Internal Medicine, Tri-Service General Hospital, National Defense Medical Center, Taipei, Taiwan; ^2^Division of Cardiology, Heart Center, Cheng Hsin General Hospital, Taipei, Taiwan; ^3^Faculty of Medicine, School of Medicine, National Yang Ming Chiao Tung University, Taipei, Taiwan; ^4^School of Public Health, National Defense Medical Center, Taipei, Taiwan; ^5^School of Medicine, National Defense Medical Center, Taipei, Taiwan; ^6^Institute of Clinical Medicine, National Yang Ming Chiao Tung University, Taipei, Taiwan

**Keywords:** proximal protection device, carotid artery stenting, stroke, carotid stenosis, occlusion intolerance

## Abstract

**Background:**

Proximal protection devices, such as the Mo.Ma system provides better neurological outcomes than the distal filter system in the carotid artery stenting (CAS) procedure. This study first evaluated the safety and efficacy of the Mo.Ma system during CAS in a single tertiary referral hospital from Taiwan. The outcomes of distal vs. proximal embolic protection devices were also studied.

**Methods:**

A total of 294 patients with carotid artery stenosis who underwent the CAS procedure were retrospectively included and divided into two groups: 152 patients in the distal filter system group and 142 patients in the Mo.Ma system. The outcomes of interest were compared between the two groups. The factors contributing to occlusion intolerance (OI) in the Mo.Ma system were evaluated.

**Results:**

The procedure success rates were more than 98% in both groups. No major stroke occurred in this study. The minor stroke rates were 2.8% (4/142) and 4.6% (7/152) in the Mo.Ma system and filter system, respectively (*p* = 0.419). Patients with hypoalbuminemia significantly predicted the risk of stroke with an odds ratio of 0.08 [95% confidence interval (CI), 0.01–0.68, *p* = 0.020] per 1 g/day of serum albumin in the filter group. A total of 12 patients developed OI in the Mo.Ma system (12/142, 8%). Low occlusion pressure predicted the occurrence of OI in the Mo.Ma group with the hazard ratios of 0.88 (95% CI: 0.82–0.96) and 0.90 (95% CI: 0.84–0.98) per 1 mmHg of occlusion systolic pressure (OSP) and diastolic pressure (ODP), respectively. We further indicated that patients with an OSP of ≥60 mmHg or an ODP of ≥44 mmHg could tolerate the procedure of occlusion time up to 400 s, while patients with an OSP of <49 mmHg or an ODP of <34 mmHg should undergo the procedure of occlusion time less than 300 s to prevent the occurrence of OI.

**Conclusion:**

We have demonstrated the safety and effectiveness of the Mo.Ma system during CAS in an Asia population. By reducing the occlusion time, our study indicated a lower risk of OI in the Mo.Ma system and proposed the optimal occlusion time according to occlusion pressure to prevent OI during the CAS procedure. Further large-scale and prospective studies are needed to verify our results.

## Introduction

Carotid artery atherosclerosis is one of the major causes of ischemic events in the cerebrovascular system and accounts for 20–30% of strokes (1, 2). Previous studies demonstrated that carotid endarterectomy (CEA) reduced the risk of stroke in patients with carotid stenosis when compared with medical treatment only (3–6). Carotid artery stenting (CAS) is a treatment alternative to CEA, which has been shown to be safe and effective when compared with CEA in the treatment of carotid artery disease (6–10). A randomized control trial compared CAS vs. CEA in asymptomatic patients with severe carotid stenosis who were not at high risk for surgical complications. The results showed that CAS was non-inferior to CEA regarding the rate of the primary composite endpoint at 1 year (event rate, 3.8 and 3.4%, respectively; *p* = 0.01 for non-inferiority). There were no significant differences in the 5-year follow-up of stroke-free survival between the CAS and CEA groups (7). The Stenting and Angioplasty with Protection of Patients with High Risk for Endarterectomy (SAPPHIRE) randomized trial was a multicenter, prospective trial conducted at 29 centers to compare clinical outcomes of CAS vs. CEA in high-surgical risk patients, which showed that CAS was non-inferior to CEA (8).

The risk of cerebral embolization is the primary concern during CAS (11). Mirco Cosottini et al. demonstrated that CAS with cerebral protection devices might reduce the number of silent ischemic lesions detected by brain magnetic resonance imaging (MRI) and diffusion-weighted imaging (DWI) (12). Embolic protection devices (EPDs), such as distal occlusion balloons, distal filters, and proximal protection devices, are encouraged to be applied to the patients during CAS to prevent cerebral embolization. A study showed that the use of EPDs was associated with a low risk of adverse events, but there was no significant difference in the risk of peri-procedural or 30-day adverse events among the different types of EPDs (13). The operators select the EPD according to the lesion morphology, vessel anatomy, and the familiarity of the devices. The combination strategy using a distal filter and proximal EPD might be a promising approach, especially in the lesions with thrombus and ulcerative lesions; however, the current evidence of such application for CAS is lacking. The Mo.Ma system is a type of proximal protection device that simultaneously blocks retrograde blood flow from the external carotid artery (ECA) and antegrade blood flow from the common carotid artery (CCA) during carotid intervention. Previous studies suggested that the Mo.Ma system significantly reduced the occurrence of new cerebral lesions and silent brain infarcts by DWI of brain MRI (14, 15), which is associated with the risk of future dementia and a steeper decline in cognitive function (16, 17). Such promising results are related to the high capacity of retrieving debris by the Mo.Ma system, instead of the distal filter system (14, 15). Moreover, the Mo.Ma system provides neuroprotective benefits throughout all phases of the procedure, including initial wire lesion crossing, while the distal filter system should cross the lesion before neuroprotection (18). The ARMOUR trial evaluated the 30-day safety and effectiveness of Mo.Ma EPD employment during the CAS procedure for high-surgical risk patients, which demonstrated that Mo.Ma proximal EPD is safe and effective for high-surgical risk patients undergoing CAS. Of note, there were no patients who suffered a symptomatic stroke during the trial (19). These results highlight the beneficial effects of the Mo.Ma system on the CAS procedure.

Current clinical studies in evaluating the clinical outcomes of the Mo.Ma system in CAS were mainly from western countries. In this study, we evaluated the safety and efficacy of Mo.Ma proximal EPD of CAS in patients with stenosis of the cervical internal carotid artery (ICA) in the Chinese population in a single tertiary referral hospital. Moreover, the predictors of occlusion intolerance (OI) as well as strategies to prevent OI in the Mo.Ma system during the CAS procedure were evaluated.

## Materials and methods

### Materials

The Institutional Review Board of Cheng Hsin General Hospital has approved this study (IRB No. 880-110-26). We included patients who received CAS in Cheng Hsin General Hospital in Taiwan from October 2008 to March 2021, which with symptomatic carotid artery stenosis with ≥60% stenosis and asymptomatic carotid artery stenosis with ≥80% stenosis based on the recommendations of CAS from The Ministry of Health and Welfare of Taiwan. Symptomatic carotid stenosis is defined as stenosis in the ICA, leading to symptoms of amaurosis fugax, transient ischemic attacks, or ischemic stroke ipsilateral to the lesion. Our study also included patients with syncope, blackouts, and fainting without other significant causes. Additionally, carotid artery stenosis following radiotherapy, carotid artery dissection resulting in stenosis or dissecting aneurysm, and patients with high CEA risks were also included. The severity of stenosis was measured by the North American Symptomatic Carotid Endarterectomy Trial (NASCET) (5) carotid stenosis measurement method, which compares the lumen size at the area with the most significant stenosis with the lumen size of the normal distal cervical ICA (5). Before carotid artery intervention, most of the patients received carotid ultrasonography (iU22 PHILIPS) and contrast-enhanced magnetic resonance angiography (MAGNETOM Aera SIEMENS), and some of the patients underwent carotid artery computed tomography angiography (SOMATOM go.Top SIEMENS). The exclusion criteria included patients who had contralateral carotid occlusion or underwent CAS with distal balloon occlusion EPD or without using EPD. The referral of patients was from cardiologists, cardiovascular surgeons, radiologists, and neurologists. Our study included all patients who received CAS with Mo.Ma proximal EPDs or distal filter EPDs. We retrospectively analyzed 142 consecutive patients who underwent CAS with Mo.Ma proximal EPDs (Invatec, Roncadelle, Italy), and 152 patients underwent CAS with distal filter EPDs (Boston Scientific Corporation, Natick, MA, USA).

### Procedure details

All the patients undergoing CAS took dual antiplatelet agents at least 7 days before the procedure. We undertook the CAS procedure mainly *via* a femoral approach or a trans-radial approach under local anesthesia. After the femoral artery or radial artery was successfully punctured, we inserted a 6F arterial sheath. Heparin was administered intravenously at a dose of 100 units/kg to maintain an activated clotting time ≥300 s. We used the diagnostic catheters Judkins left (JL) and Judkins right (JR) for coronary angiography and used the JR diagnostic catheter for selected carotid and vertebral artery angiography. We performed the target vessel angiography at the last step of diagnostic angiography and maintained the catheter at the CCA. After confirming the severity of carotid artery disease, we advanced an angled Radifocus guidewire (Terumo Corporation, Tokyo, Japan) through the JR diagnostic catheter to the ECA and then pushed the JR diagnostic catheter into the ECA. We exchanged a 0.035-inch Amplatz super-stiff wire (Cook Medical, Bloomington, IN, USA) for the angled Radifocus guidewire, and then removed the JR catheter. Subsequently, we exchanged the 9F sheath for the 6F arterial sheath and inserted the Mo.Ma catheter and then advanced it with the tip of the catheter to the proximal ECA at approximately 1 cm beyond the carotid bifurcation. Angiography was performed to confirm the proper position of the Mo.Ma system. To reduce the balloon occlusion time and prevent OI, we preloaded a 0.014” guidewire and a selected carotid stent at the level of the CCA within the Mo.Ma catheter before the balloon occlusion of ECA and CCA if we planned to deploy the stent directly. Likewise, we placed a guidewire and balloon catheter at the level of the CCA within the Mo.Ma catheter in advance if we considered the pre-dilatation of the lesion.

We inflated the balloon in the ECA of the Mo.Ma system by using 1:1 contrast medium (Visipaque 320; G.E. Healthcare, Cork, Ireland) and saline solution for obstructing the retrograde flow from the ECA to the ICA. We inflated the ECA balloon to just cover or proximal to the origin of the superior thyroid artery. Angiography was performed to confirm the complete occlusion of ECA. Subsequently, we inflated the proximal balloon in the CCA to occlude the CCA. The occlusion of ECA and CCA flow was indicated by the change in balloons’ shape from circular to cylindrical. We recorded the established back pressure value as the occlusion pressure. Meanwhile, we made an angiography to confirm the stationary flow in the CCA and ICA. Then, the guidewire crossed the lesion and was advanced to the distal extracranial ICA. We predilated the lesion with a 3.0–3.5-mm semi-complaint balloon, and the stent was deployed if the lesion was considered too tight to directly cross a stent for deployment. Subsequently, stent post-dilatation with a 5.0 or 5.5-mm Sterling balloon (Boston Scientific, Maple Grove, MN, USA) was performed. During the balloon dilatation of the carotid stenosis, the heart rate and blood pressure might be decreased due to carotid baroreceptor response. We informed the patients before the balloon inflation regarding the brief feeling of swelling or pain in the neck, and we administered atropine to block the baroreceptor response, which is effective for maintaining optimal blood pressure and heart rate during balloon dilatation. Only a minority of patients need a low dose of vasopressor dose to maintain stable hemodynamics after the procedure. Thereafter, a 30-ml Luer lock syringe aspirated the blood-containing debris through the central lumen of the Mo.Ma catheter immediately and filtered the blood through the sieves until there was no more debris. There were three aspirations with a total of 60–75 ml of blood aspirated for all our cases. We deflated the ECA balloon first, followed by the CCA balloon. We then pulled the jailed ECA balloon down to the CCA. Finally, post-stenting carotid angiography and ipsilateral intracranial angiography were performed. The femoral artery access site was closed by a vascular closure device (Angioseal™, St. Jude Medical Inc., Minnetonka, MN, USA).

### Data sources

We retrospectively reviewed the data including sex, age, baseline comorbidities, and baseline biochemistry data and reviewed the pre-procedure carotid images and the CAS video. We stratified the patients into CAS with proximal Mo.Ma EPD and distal filter EPD. The two groups of patients were compared in terms of baseline characteristics and incidence of endpoints. We also evaluated the efficacy and safety of proximal Mo.Ma EPD.

The outcomes of interest included procedural success, technical success, in-hospital stroke and death, and the rate of stroke and death at 6 months. We also assessed the balloon occlusion time, OI, and cerebral occlusion pressure of Mo.Ma proximal EPD. OI was defined as any transient neurological deficit observed during occlusion time with a complete recovery within 20 min after restoring antegrade flow (19). Occlusion time was defined as the time from inflation to deflation of the proximal balloon in the CCA (20). The definition of minor stroke was a focal neurological symptom with acute infarction per neuroimaging or clinical findings, with changes in the National Institutes of Health Stroke Scale (NIHSS) score from 1 to 4 (14). Changes in the NIHSS score of more than 4 points were classified as evidence of major stroke. The device success was defined as the ability to position, deploy and retrieve the Mo.Ma device during the index procedure. Technical success was defined as device success, including successful implantation of a carotid stent with residual stenosis <30% during the index procedure (19). Procedural success was defined as technical success without any major adverse cardiac or cerebrovascular events or unresolved antegrade flow cessation intolerance during the index procedure (19).

### Statistical analysis

Data were analyzed using R software version 3.4.4 and the statistical level was set as *p* < 0.05. The baseline characteristics were expressed as the mean ± SD or number (proportion) as appropriate, and the Student’s *t*-test and Chi-square test were used for statistical tests, respectively. The primary analysis included the risk of outcomes of interest in two groups, and the bar graphs were used to present and test by Fisher’s exact test due to the small sample size of events. We only additionally used logistic regression to analyze the risk factors for in-hospital stroke and 6-month stroke because the number of events was more than 5. Moreover, we used the Cox proportional hazard model to analyze the OI in the Mo.Ma group, which was censored at the end of the procedure to limit bias from faster cases. We also used the Kaplan–Meier curve to demonstrate the relationship between occlusion pressure and time to OI, and the tertile was used to group these patients. The Kaplan–Meier curve was based on the log-rank test, and the post hoc test was used by Bonferroni correction.

## Results

A total of 294 patients who underwent CAS were retrospectively collected. They were divided into two groups, the filter group and Mo.Ma group. Table 1 shows the basic demographic characteristics. The patients in the Mo.Ma group were younger and had a higher percentage of hyperlipidemia and cerebrovascular accident (CVA) history than the filter group. In the laboratory data, the patients in the Mo.Ma group had lower plasma uric acid and blood urea nitrogen (BUN) levels but higher plasma hemoglobin levels than the filter group. Regarding stenotic lesion characteristics, the patients in the Mo.Ma group had a high percentage of unilateral disease, but more severe stenotic lesions than the filter group.

**TABLE 1:** Corresponding characteristics in the filter and Mo.Ma groups.

Variable	Filter (*n* = 152)	Mo.Ma (*n* = 142)	*P*-value
**Demography**			
Gender (male)	115 (75.7%)	111 (78.2%)	0.610
Age (years)	71.18 ± 8.50	66.45 ± 9.42	<0.001
Smoking	59 (38.8%)	62 (43.7%)	0.399
**Disease history**			
Diabetes mellitus	66 (43.4%)	55 (38.7%)	0.414
Hypertension	126 (82.9%)	121 (85.2%)	0.588
Hyperlipidemia	99 (65.1%)	120 (84.5%)	<0.001
CVA	57 (37.5%)	74 (52.1%)	0.012
CAD	121 (79.6%)	101 (71.1%)	0.091
**Laboratory data**			
BUN (mmol/L)	3.09 ± 1.95	2.61 ± 1.53	0.021
Creatinine (mmol/L)	0.11 ± 0.07	0.1 ± 0.08	0.199
GOT (U/L)	26.72 ± 13.23	26.12 ± 13.58	0.703
GPT (U/L)	24.62 ± 13.92	26.50 ± 16.75	0.296
HDL (mmol/L)	1.02 ± 0.28	1.06 ± 0.26	0.177
LDL (mmol/L)	2.42 ± 0.79	2.32 ± 0.82	0.310
Uric acid (mmol/L)	0.38 ± 0.12	0.34 ± 0.08	0.001
Triglyceride (mmol/L)	1.49 ± 0.75	1.43 ± 0.95	0.558
Albumin (mmol/L)	0.53 ± 0.06	0.53 ± 0.05	0.998
Glucose AC (mmol/L)	6.43 ± 2.63	6.18 ± 2.18	0.361
WBC (10^9^/L)	6.73 ± 1.73	6.87 ± 1.99	0.516
Hemoglobin (g/L)	129.90 ± 18.60	134.50 ± 15.50	0.021
Hematocrit (L/L)	0.39 ± 0.08	0.4 ± 0.04	0.347
Platelet (10^9^/L)	209.91 ± 52.81	220.13 ± 53.72	0.101
**Other**			
Symptom	116 (76.3%)	102 (71.8%)	0.183
Unilateral/bilateral stenosis			0.018
Unilateral	99 (65.1%)	111 (78.2%)	
Bilateral	51 (33.6%)	31 (21.8%)	
Collateral flow *via* Acom	70 (46.1%)	80 (56.3%)	0.078
Mean stenosis (%)	83.57 ± 7.91	86.05 ± 7.58	0.007
Mean contralateral stenosis (%)	24.28 ± 35.99	12.36 ± 26.13	0.001
Pre-dilatation	69 (45.4%)	67 (47.2%)	0.759
Post-dilatation	145 (95.4%)	141 (99.3%)	0.068
Calcified lesions	35 (23.0%)	28 (19.7%)	0.490
Transradial approach	9 (11.1%)	0 (0.0%)	<0.001

Acom, anterior communicating artery; BUN, blood urea nitrogen; CAD, coronary artery disease; CVA, cerebrovascular accident; GOT, aspartate aminotransferase; GPT, alanine aminotransferase; Glucose AC, glucose ante cibum; HDL, high-density lipoprotein-cholesterol; LDL, low-density lipoprotein-cholesterol; stenosis (%), percentage of stenosis in lesion site before dilatation; WBC, white cell count.

**FIGURE 1:**
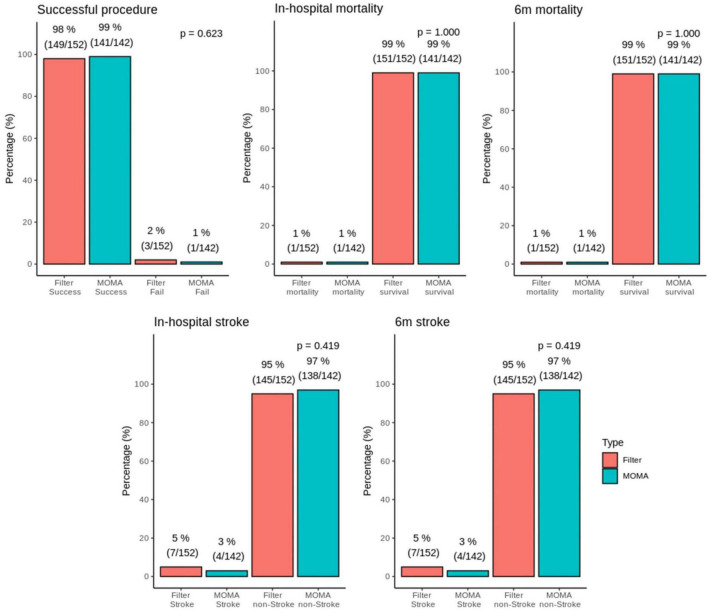
Bar graphs of successful surgery, in-hospital mortality, 6 months mortality, stroke, and 6 months stroke in the MOMA and filter groups. There was no statistical difference in in-hospital stroke and 6 months stroke between the Mo.Ma group and filter group.

There were three and one failed procedures in the filter and Mo.Ma groups, respectively (Figure 1). One patient died after the procedure in the filter group due to receiving coronary artery bypass grafting (CABG) surgery with cardiogenic shock. There was also a mortality case in the Mo.Ma group with the same cause of death. No patients suffered from a major stroke in the study. However, 2.8% (4/142) of patients had a minor stroke in the Mo.Ma group, which was lower than that in the filter group (4.6%, 7/152), although the difference was not statistically significant. As shown in Table 2, patients with hypoalbuminemia had a significantly (*p* = 0.020) higher risk of stroke within 6 months, and the odds ratio was 0.08 (95% CI: 0.01–0.68) per 1 g/dl of serum albumin. Table 3 shows that no significant factors contributed to stroke within 6 months in the Mo.Ma group. The other characteristics were not associated with outcomes of interest, including successful procedure, in-hospital mortality, and mortality within 6 months (data not shown). Because the serum albumin concentrations were similar (*p* = 0.825) in the filter group (3.56 ± 0.39) and Mo.Ma group (3.55 ± 0.33), there were no potential confounding factors in further outcomes of interest comparison between these two groups.

**TABLE 2:** Adjusted odds ratios (and 95% CIs) on in-hospital stroke and 6 months stroke in filter group.

Variable	In-hospital stroke OR (95% CI)^#^	*P*-value	6 months stroke OR (95% CI)^#^	*P*-value
**Demography**				
Smoking	2.10 (0.42–10.64)	0.368	2.10 (0.42–10.64)	0.368
**Disease history**				
Hypertension	1.04 (0.12–8.94)	0.973	1.04 (0.12–8.94)	0.973
Diabetes mellitus	1.08 (0.23–5.01)	0.926	1.08 (0.23–5.01)	0.926
Hyperlipidemia	0.51 (0.11–2.38)	0.393	0.51 (0.11–2.38)	0.393
Previous CVA	0.47 (0.09–2.49)	0.376	0.47 (0.09–2.49)	0.376
CAD	1.71 (0.20–14.55)	0.625	1.71 (0.20–14.55)	0.625
**Laboratory data**				
BUN	0.99 (0.92–1.07)	0.849	0.99 (0.92–1.07)	0.849
Creatinine	1.09 (0.63–1.90)	0.749	1.09 (0.63–1.90)	0.749
GOT	0.99 (0.92–1.07)	0.841	0.99 (0.92–1.07)	0.841
GPT	0.98 (0.91–1.06)	0.601	0.98 (0.91–1.06)	0.601
HDL	0.98 (0.90–1.06)	0.547	0.98 (0.90–1.06)	0.547
LDL	1.00 (0.98–1.03)	0.918	1.00 (0.98–1.03)	0.918
Uric acid	0.90 (0.56–1.45)	0.663	0.90 (0.56–1.45)	0.663
Triglyceride	1.00 (0.98–1.01)	0.654	1.00 (0.98–1.01)	0.654
Albumin	0.08 (0.01–0.68)	0.020	0.08 (0.01–0.68)	0.020
Glucose AC	1.00 (0.97–1.02)	0.731	1.00 (0.97–1.02)	0.731
WBC	1.00 (1.00–1.00)	0.509	1.00 (1.00–1.00)	0.509
Hemoglobin	0.84 (0.53–1.31)	0.434	0.84 (0.53–1.31)	0.434
Hematocrit	0.96 (0.86–1.08)	0.508	0.96 (0.86–1.08)	0.508
Platelet	1.00 (1.00–1.00)	0.763	1.00 (1.00–1.00)	0.763
**Other**				
Pre-dilatation	0.16 (0.02–1.36)	0.092	0.16 (0.02–1.36)	0.092
Calcified lesions	0.00 (0.00–∞)	0.994	0.00 (0.00–∞)	0.994
Collateral flow *via* Acom	0.15 (0.02–1.25)	0.079	0.15 (0.02–1.25)	0.079

^#^All result of Adj-OR were adjusted by sex, age. Acom, anterior communicating artery; BUN, blood urea nitrogen; CAD, coronary artery disease; CVA, cerebrovascular accident; GOT, aspartate aminotransferase; GPT, alanine aminotransferase; Glucose AC, glucose ante cibum; HDL, high-density lipoprotein-cholesterol; LDL, low-density lipoprotein-cholesterol; WBC, white cell count.

**TABLE 3:** Adjusted odds ratios (and 95% CIs) on in-hospital stroke and 6 months stroke in the Mo.Ma group.

Variable	In-hospital stroke OR (95% CI)^#^	*P*-value	6 months stroke OR (95% CI)^#^	*P*-value
Demography	0.39 (0.04–4.04)	0.429	0.39 (0.04–4.04)	0.429
**Smoking**				
**Disease history**				
Hypertension	∞ (0.00–∞)	0.996	∞ (0.00–∞)	0.996
Diabetes mellitus	0.00 (0.00–∞)	0.996	0.00 (0.00–∞)	0.996
Hyperlipidemia	∞ (0.00–∞)	0.996	∞ (0.00–∞)	0.996
Previous CVA	0.75 (0.10–5.65)	0.781	0.75 (0.10–5.65)	0.781
CAD	∞ (0.00–∞)	0.997	∞ (0.00–∞)	0.997
**Laboratory data**				
BUN	0.83 (0.61–1.12)	0.215	0.83 (0.61–1.12)	0.215
Creatinine	0.06 (0.00–9.89)	0.274	0.06 (0.00–9.89)	0.274
GOT	0.88 (0.72–1.08)	0.231	0.88 (0.72–1.08)	0.231
GPT	0.91 (0.78–1.07)	0.261	0.91 (0.78–1.07)	0.261
HDL	0.93 (0.79–1.09)	0.355	0.93 (0.79–1.09)	0.355
LDL	1.03 (1.00–1.06)	0.067	1.03 (1.00–1.06)	0.067
Uric acid	0.92 (0.43–1.94)	0.819	0.92 (0.43–1.94)	0.819
Triglyceride	1.00 (0.99–1.01)	0.922	1.00 (0.99–1.01)	0.922
Albumin	14.45 (0.49–424.05)	0.121	14.45 (0.49–424.05)	0.121
Glucose AC	0.98 (0.94–1.03)	0.525	0.98 (0.94–1.03)	0.525
WBC	1.00 (1.00–1.00)	0.335	1.00 (1.00–1.00)	0.335
Hemoglobin	1.60 (0.80–3.19)	0.183	1.60 (0.80–3.19)	0.183
Hematocrit	1.23 (0.95–1.60)	0.121	1.23 (0.95–1.60)	0.121
Platelet	1.00 (1.00–1.00)	0.464	1.00 (1.00–1.00)	0.464
**Other**				
Pre-dilatation	3.33 (0.33–33.48)	0.306	3.33 (0.33–33.48)	0.306
Calcified lesions	0.00 (0.00–∞)	0.996	0.00 (0.00–∞)	0.996
Collateral flow *via* Acom	0.21 (0.02–2.13)	0.184	0.21 (0.02–2.13)	0.184

^#^All results of Adj-OR were adjusted by sex, age. Acom, anterior communicating artery; BUN, blood urea nitrogen; CAD, coronary artery disease; CVA, cerebrovascular accident; GOT, aspartate aminotransferase; GPT, alanine aminotransferase; Glucose AC, glucose ante cibum; HDL, high-density lipoprotein-cholesterol; LDL, low-density lipoprotein-cholesterol; WBC, white cell count.

**FIGURE 2:**
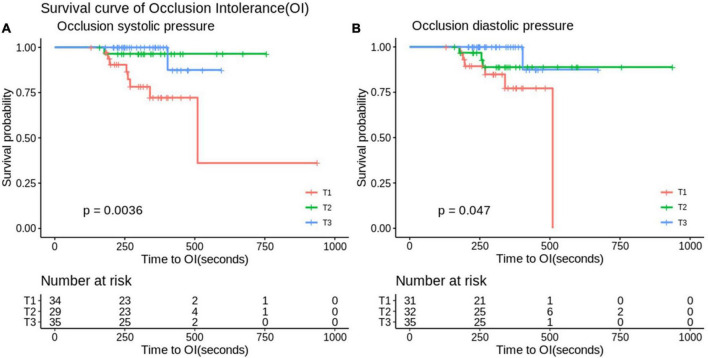
Analyze the survival curve of occlusion systolic **(A)** and diastolic **(B)** pressure stratification on time to occlusion intolerance (OI). In the Mo.Ma group, there were significant differences between OI in the stratification of occlusion diastolic and systolic pressure (*p* < 0.05). Systolic pressure: Q1, systolic < 49; Q2, 60 > systolic ≥ 49; Q3, systolic ≥ 60 (mmHg). Diastolic pressure: Q1, diastolic < 34; Q2, 44 > diastolic ≥ 34; Q3, diastolic ≥ 44 (mmHg).

In the application of the Mo.Ma EPD system, the procedure time is an important issue because the stationary carotid blood flow may result in OI during the CAS procedure. There were a total of 12 patients who developed OI in our study (12/142, 8%, data not shown). We then evaluated the factors contributing to OI (Table 4). All 12 patients who developed OI in the study had a history of hyperlipidemia, resulting in the extremely high hazard ratio (HR) of hyperlipidemia to the risk of OI. By Cox proportional hazard model analysis, we found that low occlusion systolic and diastolic pressure (ODP) significantly predicted the occurrence of OI, instead of the absence of anterior communicating artery (Acom) collateral flow. No other significant factors contributed to the occurrence of OI in the Mo.Ma group. The hazard ratios of occlusion systolic pressure (OSP) and ODP per 1 mmHg were 0.88 (95% CI: 0.82–0.96) and 0.90 (95% CI: 0.84–0.98), respectively. We further categorized the three tertile groups by the values of OSP and ODP and presented the time-events curve in Figure 2. The *post hoc* test shows that the risk of T1 was significantly higher than T2 (*p* = 0.023) and T3 (*p* = 0.023) in OSP analysis, but the difference between T2 and T3 was not significant (*p* = 0.937). Moreover, the results of the pairwise *post hoc* test in occlusion ODP analysis were not significant (p. T1 vs. T2 = 0.213; p. T1 vs. T3 = 0.067; p. T2 vs. T3 = 0.328). Collectively, the T1 group shows higher risk of OI compared to the patients with higher occlusion pressure. There were no OI events before the first 200 s, and more than 20% of patients with systolic pressure of <49 mmHg presented with OI within 250 s. In contrast, the first event was presented at 400 s in patients with an OSP of ≥60 mmHg. Importantly, all patients with an occlusion ODP of <34 mmHg presented OI after 520 s, which provides the recommended procedure time for patients with different OSPs and ODPs. All the results proposed that patients with OSP of ≥60 mmHg or occlusion ODP of ≥44 mmHg could tolerate the procedure of occlusion time up to 400 s, while patients with OSP of <49 mmHg or occlusion ODP of <34 mmHg should undergo the procedure of occlusion time less than 300 s to prevent the occurrence of OI.

**TABLE 4:** Cox proportional analysis on time to occlusion intolerance (OI) in the Mo.Ma group.

Variable	HR (95% CI)^#^	*P*-value
**Demography**		
Smoking	0.79 (0.23–2.75)	0.709
**Disease history**		
Hypertension	0.99 (0.21–4.63)	0.987
Diabetes mellitus	3.05 (0.90–10.34)	0.073
Hyperlipidemia	∞ (0.00–∞)	0.998
Previous CVA	0.88 (0.28–2.76)	0.827
CAD	0.75 (0.21–2.62)	0.650
**Laboratory data**		
BUN	1.00 (0.95–1.06)	0.902
Creatinine	0.30 (0.02–3.59)	0.339
GOT	0.96 (0.88–1.05)	0.404
GPT	0.98 (0.93–1.04)	0.516
HDL	1.02 (0.97–1.08)	0.425
LDL	0.98 (0.96–1.01)	0.173
Uric acid	0.69 (0.42–1.14)	0.147
Triglyceride	1.00 (0.99–1.01)	0.734
Albumin	1.10 (0.18–6.62)	0.920
Glucose AC	1.01 (1.00–1.02)	0.083
WBC	1.00 (1.00–1.00)	0.322
Hemoglobin	1.20 (0.80–1.80)	0.389
Hematocrit	1.04 (0.90–1.21)	0.589
Platelet	1.00 (1.00–1.00)	0.377
**Procedure**		
Occlusion systolic pressure	0.88 (0.82–0.96)	0.002
Occlusion diastolic pressure	0.90 (0.84–0.98)	0.009
Bilateral stenosis	2.47 (0.76–8.04)	0.134
Pre-dilatation	0.76 (0.24–2.39)	0.643
Post-dilatation	∞ (0.00–∞)	0.998
Calcified lesions	1.26 (0.34–4.69)	0.734
Collateral flow *via* Acom	1.09 (0.34–3.48)	0.883

^#^All results of Adj-OR were adjusted by sex, age. Acom, anterior communicating artery; BUN, blood urea nitrogen; CAD, coronary artery disease; CVA, cerebrovascular accident; GOT, aspartate aminotransferase; GPT, alanine aminotransferase; Glucose AC, glucose ante cibum; HDL, high-density lipoprotein-cholesterol; LDL, low-density lipoprotein-cholesterol; WBC, white cell count.

## Discussion

By retrospective analysis of cohort data from a tertiary referring hospital, we demonstrated that the Mo.Ma system is non-inferior to the distal filter system regarding the procedure success rate, stroke, and mortality in patients with carotid artery stenosis. Although it may result from the respective design without randomization, we adjusted the factors of gender and age in the further analysis. Hypoalbuminemia significantly predicts the risk of 6-month stroke among patients undergoing distal filter procedures. Importantly, in the Mo.Ma group, patients with low occlusion systolic or ODP had a higher risk of OI than those with high occlusion systolic or ODP. The analysis of the time-events curve of OI clearly indicated that patients in the group of optimal high OSP or ODP could have more occlusion time of up to 400 s to finish the procedure. However, the occlusion time should be shortened to less than 300 s in patients with OSP of <49 mmHg or occlusion ODP of <34 mmHg to prevent the occurrence of OI.

The procedure success rates were more than 98% in both the filter and Mo.Ma systems in our study with stroke rates of 4.6 and 2.8% in the filter system and Mo.Ma system, respectively, which is compatible with previous randomized control trials for CAS (7). Although one death occurred in both the filter and Mo.Ma systems, the causes of death were related to the subsequent CABG procedure, instead of the CAS procedure. Importantly, all the stroke cases were minor strokes, nearly to TIA without major stroke, whose symptoms completely resolved before discharge. Such results highlighted the safety and effectiveness of the CAS procedure in our study, regardless of the distal filter system or Mo.Ma system.

We showed that the group of systolic occlusion pressure <49 or diastolic <34 mmHg significantly predicts the occurrence of OI, which is consistent with a previous study, indicating that low occlusion pressure is associated with the risk of OI (18). In the study from Kwon et al., the cutoff value was systolic occlusion pressure ≦42 mmHg with a sensitivity of 74% and specificity of 73% for OI (21). Giugliano et al. proposed that a mean occlusion pressure ≦40 mmHg provides a sensitivity of 68.5% and specificity of 93.3% for predicting OI (18). In another clinical setting as CEA, the validated threshold for selective shunting was 40 mmHg for mean stump pressure for the prevention of OI (22). All the results established a significant predictor of low occlusion pressure for OI in the Mo.Ma system. It has been suggested that the occurrence of OI is more frequent in patients with hypertension (18), probably due to their higher cerebral perfusion pressures. Moreover, the factors of the absence of Acom collateral flow protection (23), contralateral carotid occlusion (18), and absence of tight stenosis of the target vessel (18) have been suggested to significantly predict the risk of OI. In our study, contralateral carotid occlusion was the study exclusion criterion. Hypertension and absence of collateral flow *via* Acom and tight stenosis of the target vessel were not predictors for OI. Such discrepancies may be due to different study populations, which need investigations with multicenter and randomized control trials.

We found that patients with high occlusion pressure (OSP of ≥60 mmHg or occlusion ODP of ≥44 mmHg) could tolerate the procedure of occlusion time up to 400 s without the occurrence of OI, while more than 20% of patients with low occlusion pressure (OSP of <49 mmHg or occlusion ODP of <34 mmHg) presented with OI at the occlusion time within 250 s. A study from Giugliano et al. suggested that in the condition of mean occlusion pressure above 40 mmHg, the group with an occlusion time of more than 300 s was significantly associated with OI compared to the group with an occlusion time of less than 300 s (18). Interestingly, they indicated that in the condition of occlusion pressure less than 40 mmHg, an occlusion time of more than 300 s did not predict the risk of OI. Both studies clearly established the roles of occlusion pressure and occlusion time on the risk of OI. A previous study suggested that dealing with OI during the procedure includes breaking the procedure into stages, comprising of initial lesion wiring and balloon pre-dilatation, stent placement, balloon post-dilatation, and aspiration. These steps can be completed in stages according to the patient’s tolerability to the flow reversal or flow cessation (24). Expediting the procedure in high-risk patients to reduce the occlusion time reasonably ameliorates the neurological symptoms of patients with the interrupted antegrade flow. Our promising and unique approaches involve preloading the guidewire, balloon, or stent in the distal part of the Mo.Ma catheter before the ECA and CCA balloon occlusion. After achieving the flow reversal or flow cessation, the wire could immediately cross the lesion, followed by balloon dilatation or stent implantation. This approach effectively reduced the occlusion time in the procedure of Mo.Ma system-assisted CAS. Such effects partially explained the low incidence of OI in our study compared with previous studies (8 vs. 13.8%) (24). Of note, we further provided the practical occlusion time according to the level of occlusion pressure to prevent the occurrence of OI.

Previous studies have shed light on possible mechanisms linking occlusion pressure and the occurrence of OI. Occlusion pressure, measured in the ICA after the inflation of the balloons in the common and external carotid arteries, is represented as the perfusion pressure. The measurement of distal ICA pressure has been applied to predict ischemia in the procedures of CEA or permanent occlusion of the ICA for the treatment of complex cerebral aneurysms (25). Low perfusion pressure could result from the abnormal structure or obstructions of the Circle of Willis (21, 26), the presence of contralateral carotid occlusion (18, 27), or the drop in arterial blood pressure (22, 28). Moreover, studies from Giugliano et al. suggested that hypercholesterolemia is a significant predictor of occlusion pressure less than 40 mmHg (18). These elements could be attributed to inadequate compensating flow to the ipsilateral cerebral perfusion, thus resulting in hypoperfusion, followed by the occurrence of OI (22).

In our study, the average plasma albumin levels were 0.53 mmol/L, which is in the low limit of normal ranges (0.53–0.83 mmol/L). There is no statistical significance in plasma albumin levels between the filter group and Mo.Ma group (Table 1). By logistic regression analysis, we found that plasma albumin levels could not predict the risks of in-hospital or 6 months stroke (Table 3). Interestingly, we found that hypoalbuminemia significantly predicts the peri-procedural TIA in the filter group. Previous studies suggested that a low serum albumin concentration was an independent predictor of incident cardioembolic and cryptogenic stroke among 2,986 patients free of stroke for 12 years (29). Malnutrition and inflammation with enhanced oxidative stress and augmented platelet aggregation possibly contribute to the occurrence of stroke in patients with hypoalbuminemia. A study from Hung et al. suggested that a history of diabetes, an ICA to CCA ratio below 0.7, and a low stent to CCA ratio were significantly associated with peri-procedural neurological complications by multivariate analysis (30). Possible reasons underlying the discrepancies include different study populations, operators, and procedure protocols.

There are some limitations in our study. First, this is a single-center retrospective study, and potential bias could not be completely controlled. Moreover, the limited patient population might be a concern for outcome analysis. Second, the CAS procedure is operator-dependent. The experience of an interventionalist significantly affects the procedure outcome, which cannot be extrapolated to all patients. Third, the lesion complexities and plaque morphologies, which might influence the outcomes, were not evaluated in the study. Fourth, DWI of brain MRI to detect silent brain infarcts was not performed. Fifth, in a systemic review, the rates of in-stent restenosis >50% are 3.9 and 5.7% at 6 and 12 months after CAS, respectively. A recent meta-analysis showed that CAS had a higher risk of restenosis (>50%) than CEA and a similar risk of severe restenosis (>70%) with CEA (31). Our study did not measure the rate of in-stent restenosis at 6 or 12 months due to the low prevalence of in-stent restenosis and ethical issues with the risk of the repeated procedure. Sixth, most patients received clinical follow-up after CAS with the resolution of the initial symptoms of carotid artery stenosis. However, the current study did not compare the symptoms between the two groups before and after CAS. Moreover, except for body mass index (BMI) and plasma albumin levels, other information regarding nutritional status is not available in the study. Finally, we did not investigate the safety and efficacy of other EPDs in the study. Even with these limitations, our study provides novel and critical information regarding the application of the Mo.Ma system to a Chinese population for those interventionalists who perform CAS.

Collectively, our study proposed the safety and efficacy profiles of the Mo.Ma system in a Chinese population. We showed that compared with the distal filter system, the Mo.Ma system exhibited compatible performance in successful procedures, stroke, and mortality. Most importantly, low occlusion systolic or ODP was the single significant factor predicting the risk of OI among patients undergoing the Mo.Ma procedure. Although further prospective and large-scale studies are needed to prove our results, we first recommended that patients with OSP of ≥60 mmHg or occlusion ODP of ≥44 mmHg could tolerate the occlusion time of 400 s, while patients with OSP of <49 mmHg or occlusion ODP of <34 mmHg should reduce the occlusion time to less than 300 s to prevent the occurrence of OI.

## Data availability statement

The original contributions presented in this study are included in the article/supplementary material, further inquiries can be directed to the corresponding author.

## Ethics statement

The studies involving human participants were reviewed and approved by the Institutional Review Board of Cheng Hsin General Hospital (IRB No. 880-110-26). Written informed consent for participation was not required for this study in accordance with the national legislation and the institutional requirements.

## Author contributions

C-CC, C-SL, Y-FL, and T-PT participated in the research design and wrote the manuscript. T-PT performed the experiments and was the principal investigator of this project. CL performed the statistical analysis. All authors interpreted the data, contributed to the editing of the manuscript, and gave the final approval of this manuscript.
